# Diversity Analysis of Fruit Phenotypic Traits in *Camellia reticulata*

**DOI:** 10.3390/plants15050771

**Published:** 2026-03-03

**Authors:** Yujia Zeng, Hongxing Xiao, Fujun Yan, Xinran Yang, Xueqin Wu, Yuanyuan Huang, Wei Zheng, Yunlong Wu, Baolin Liang, Zhonglang Wang, Fang Geng

**Affiliations:** 1 College of Landscape Architecture and Horticulture, Southwest Forestry University, Kunming 650224, China; 18287788533@163.com (Y.Z.); hongxing1222025@163.com (H.X.); yfj940919436@163.com (F.Y.); 15287878221@163.com (X.Y.); 15750152833@163.com (X.W.); 13698707504@163.com (Y.H.); zhengw@swfu.edu.cn (W.Z.); 2 Kunming Yiliang Zhengyang Camellia Garden Afforestation Co., Ltd., Kunming 652199, China; csfugf@163.com; 3 Property Management Department of the Assets Management Company, Southwest Forestry University, Kunming 650224, China; 17387171541@163.com; 4 Kunming Institute of Botany, Chinese Academy of Sciences, Kunming 650201, China; wang@mail.kib.ac.cn

**Keywords:** *Camellia reticulata*, populations, cultivars, fruit, phenotypic diversity

## Abstract

*Camellia reticulata* is a valuable woody species prized for both its ornamental and oil-producing qualities. This study focused on four qualitative traits and nine quantitative traits of the fruits collected from nine wild populations and 30 cultivated varieties of *C. reticulata*. Multivariate statistical methods were employed to analyze the variation patterns of these fruits among populations and varieties, aiming to provide a scientific basis for the resource utilization and genetic improvement of this species. The results showed that the pericarp color clustered into two series: an orange-yellow (red) series (found in eight populations and all 30 cultivars) and a yellow-green series (unique to the Heiniu Mountain I population). The a* value was identified as the key indicator for distinguishing between these two color-series. The fruit shape was predominantly spherical, the seed shape was mostly hemispherical, and the seed coat color was primarily brown. Significant differences (*p* < 0.05) were observed among the nine quantitative phenotypic traits. Fruit weight exhibited the greatest variation (ranging from 28.499 g to 149.068 g), with particularly prominent differences among populations (Fengqing I was the heaviest at 149.068 g, while Yongping I was the lightest at 28.499 g). The coefficients of variation (*CV*) for phenotypic traits within populations ranged from 17.209% to 60.803% (mean 31.655%), and within varieties from 13.951% to 72.911% (mean 35.290%). Based on *CV* weights, seed weight showed the largest variation amplitude (21.342%) among populations, while seed number showed the largest variation amplitude (22.956%) among varieties. Correlation analysis revealed that all nine traits exhibited highly significant correlations across different populations and cultivars. Principal component analysis (PCA) indicated that the eigenvalues of the first two principal components were both greater than 1.00, with cumulative contribution rates reaching 73.570% for populations and 76.064% for cultivars, respectively. Cluster analysis grouped the studied materials into three clusters. The comprehensive evaluation identified the cultivar ‘Lichan’ as having the optimal performance (F = 2.410). Box plots revealed greater dispersion in seed number and pericarp thickness within wild populations, while cultivated varieties showed a wider distribution in locule number and fruit transverse diameter. Frequency distribution analysis demonstrated that all traits followed a normal distribution (R^2^ = 0.673~0.999). Among them, fresh seed weight and fruit transverse diameter displayed obvious skewness. Furthermore, the variation in seed number was significantly higher in wild populations than in cultivars. This study reveals rich phenotypic variation in fruit traits between wild populations and cultivated groups of *C. reticulata*, with fruit size and seed number identified as key traits. These findings provide an important basis for the subsequent selection of hybrid parents and breeding of high-yield, high-oil varieties.

## 1. Introduction

*Camellia reticulata* belongs to the Camellia section of the genus *Camellia* within the family Theaceae. It boasts a wide variety of cultivars, characterized by large and brightly colored flowers, lush foliage, and an elegant tree form, granting it exceptionally high ornamental value [1,2,3,4]. Simultaneously, it serves as an important woody oil species in China, offering significant ecological benefits and economic value, making it an outstanding tree species with comprehensive advantages [5]. With a cultivation history of 1500 years, *C. reticulata* is primarily distributed in Yunnan, southwestern Sichuan, and western Guizhou. Notably, regions such as Chuxiong, Tengchong, and Dali in Yunnan host extensive primitive wild communities. Influenced by factors such as natural hybridization, artificial selection, and environmental conditions, these populations have developed into germplasm resources rich in genetic variation [6,7]. As of November 2025, according to statistics from the International Camellia Register (https://camellia.iflora.cn/), the number of registered cultivars of *C. reticulata* has reached 859.

In germplasm resource evaluation, phenotypic trait analysis serves as the most direct and straightforward method for detecting genetic variation, and plays a key role in resource identification and conservation [8,9]. Among these, research on the genetic diversity of fruit characteristics is particularly important, as it forms the foundation for the direct application and innovation of germplasm resources. This method has been widely applied in various economic plants such as *Diospyros kaki* [10], peras (*Pyrus* L.) [11], and cherry (*Cerasus pseudocerasus*) [12], providing a theoretical basis for their genetic improvement. Furthermore, studies indicate that significant differentiation exists in fruit phenotypic traits between cultivated varieties and wild populations: cultivated varieties typically exhibit traits that align with human utilization needs, such as increased fruit size and thinner pericarp, while wild populations tend to retain natural adaptive characteristics, including smaller fruit size and thicker pericarp [13,14,15]. Most species in the genus *Camellia* are cross-pollinated and commonly exhibit reproductive barriers such as low self-compatibility and “more flowers but fewer fruits,” which not only limits yield but also significantly affects their genetic structure and phenotypic variation [16]. In oil-tea camellia breeding practice, traits such as large fruit size, thin pericarp, and high seed yield have consistently been the main target traits for cultivar selection [17]. Currently, fruit phenotypic traits of *C. chekiangoleosa* [18,19], *C. meiocarpa* [20], and *C. oleifera* [14] have been extensively studied. However, research on *C. reticulata* has primarily focused on genetic diversity [21], population structure [22], floral morphology [23], leaf characteristics [24], cultivar selection [25], and seed germination traits [26,27,28]. There remains a relative lack of research on the patterns of fruit morphological variation and their evolutionary trends in this species, and the characteristics of its phenotypic variation are still not well understood. Therefore, this study collected fruits from nine natural populations and thirty cultivated varieties of *C. reticulata*. Using methods including significance testing, correlation analysis, and principal component analysis, we systematically evaluated the patterns of phenotypic trait variation and elucidated the distinguishing characteristics between the two types of populations. The aim is to provide theoretical support for the breeding of superior varieties and the production of high-quality tea oil from this species.

## 2. Results

### 2.1. Analysis of Differences in Quantitative Phenotypic Traits of Fruits

The mean values and standard deviations of the quantitative phenotypic traits of fruits from nine populations and thirty cultivars of *C. reticulata* were compared for differences (Table S1). The results indicated significant differences (*p* < 0.05) in most fruit phenotypic traits among the studied materials. Fruit weight exhibited the most pronounced variation, with mean values ranging from 28.499 to 149.068 g. The extreme values were both from different populations. For instance, the mean FW of the Fengqing I population reached 149.068 g, which was significantly higher than that of other samples, while the Yongping I population had the lowest mean FW at only 28.499 g. Significant differences were also observed in FL and FH, with mean values ranging from 41.032 to 72.929 mm and 26.654 to 60.534 mm, respectively. Among all samples, the cultivar ‘Kunming Chun’ had the largest mean FL, and the Fengqing I population had the largest mean FH. In contrast, the mean values for both FL and FH of the Yongping I population were significantly lower than those of the other samples.

Traits including PT, SW, SL, SH, and SN also showed significant variation. Their mean value ranges were 4.723–16.115 mm, 3.157–28.204 g, 12.653–21.192 mm, 15.052–24.630 mm, and 1.450–11.800 seeds, respectively. Regarding ON, the difference was relatively small, with mean values ranging from 1.350 to 4.600. The cultivar ‘Donglin’ had the highest mean ON at 4.600, which was significantly greater than other samples, whereas the cultivar ‘Duxin Shizitou’ had a significantly lower number compared to others. The results demonstrate clear differences in the nine studied phenotypic traits of fruits among the populations and cultivars of *C. reticulata*. FW exhibited the greatest variability, while ON showed the smallest range of variation, indicating that FW is the most variable trait, whereas the number of locules is relatively stable.

### 2.2. Analysis of Phenotypic Differences in Fruit Color

For the nine populations of *C. reticulata*, the fruit L* values ranged from 45.735 to 66.594, a* values from −1.531 to 11.358, and b* values from 27.200 to 38.465. The *h°* values range from −0.611 to 1.340, and C* values from 28.531 to 38.925. There were minor differences in pericarp color parameters among the different populations. Significant differences (*p* < 0.05) in L*, a*, b*, *h°*, and C* values were found only between the samples collected from Heiniu Mountain I and the other populations (Table S2). Furthermore, the fruit color across the nine populations showed a continuous variation from yellowish-green (negative a* value) to orange-red (positive a* value). As the red tone (a*) intensified, the yellow tone (b*) also deepened correspondingly, resulting in a gradual shift in fruit color from yellowish-green to orange-red.

The fruit L* value among the 30 cultivars of *C. reticulata* ranged from 38.784 to 51.825, the a* value from 5.763 to 13.490, the b* value from 23.271 to 38.126, the *h°* value from 1.165 to 1.384, and the C* values from 24.454 to 39.480. Significant differences (*p* < 0.05) were observed in pericarp color parameters among the cultivars (Table S2). The lightness (L*) of ‘Yulan Cha’ and ‘Lifang’ was significantly higher than that of other cultivars. The a* value of ‘Xuejiao’ and ‘Duxin Shizitou’ were significantly higher, while the differences in a* values among most cultivars were not significant. The b* value of ‘Lifang’ was significantly elevated, whereas that of ‘Jingan Cha’ was significantly lower. The *h°* value showed relatively minor variation across cultivars. Furthermore, ‘Lifang’ exhibited the highest C* value, which was significantly greater than the others, while ‘Jingan Cha’ had the significantly lowest C* value. The fruit color of different cultivars predominantly showed a continuous transitional characteristic from orange-red (with low a* and b* values) to orange-yellow (with high a* and b* values), typically driven by a concurrent increase in both the a* value (indicating enhanced red tone) and the b* value (indicating deepened yellow tone).

**Figure 1:**
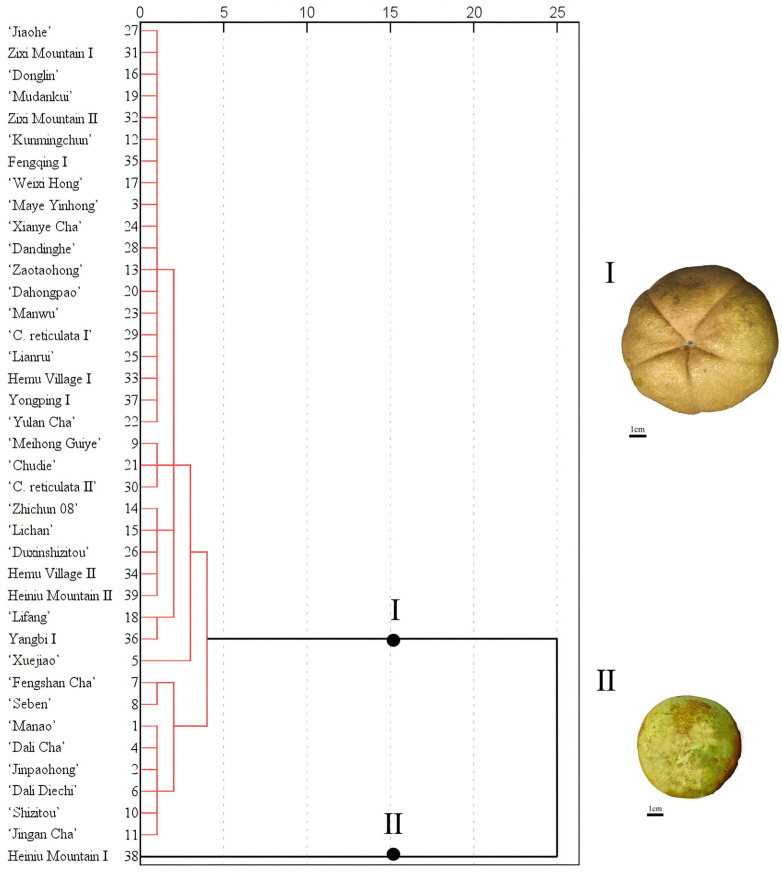
Clustering of *Camellia reticulata* fruit colors based on L*, a*, and b* values. I: orange-yellow (red) series, represented by the *C. reticulata* ‘Donglin’; II: yellow-green series, represented by *C. reticulata* from Heiniu Mountain, Chuxiong.

**Figure 2:**
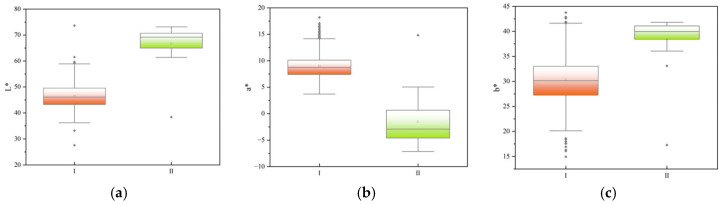
Box plots of different fruit colors of *C. reticulata*. (**a**): L* value. (**b**): a* value. (**c**): b* value. I: orange-yellow (red) series, II: yellow-green series.

Based on the cluster analysis of L*, a*, and b* values from the pericarps of nine populations and 30 cultivars, the fruit color phenotypes can be divided into two distinctly different groups at a Euclidean distance of 15 (Figure 1). Group I belongs to the orange-yellow (red) color series, encompassing the fruits of 8 populations and all 30 cultivars. Group II is the yellow-green series, containing only the Heiniu Mountain I population. Comparative analysis using box plots of the L*, a*, and b* values revealed significant differences in color parameters between the two-color series: as the fruit color shifts from yellow-green to orange-yellow, the lightness (L*) shows a gradual decreasing trend. In terms of a* value distribution, the yellow-green series has the smallest variation range. Although it partially overlaps with the distribution of the orange-yellow (red) series, the a* value can serve as a key indicator for distinguishing the two series. Conversely, the distribution of b* values indicates that the range for the yellow-green series is completely encompassed within the distribution range of the orange-yellow (red) series (Figure 2).

### 2.3. Analysis of Diversity in Fruit Qualitative Traits Among Different Populations and Cultivated Varieties

**Figure 3:**
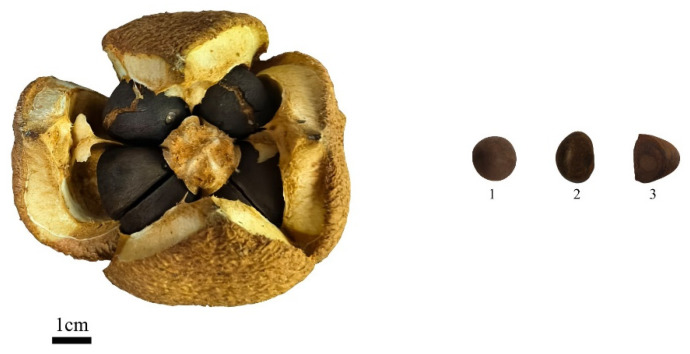
Example of fruit seed shape. Take the *C. reticulata* ‘Dali Diechi’ as an example: 1: spherical; 2: hemispherical; 3: conical.

According to Table 1 and Table 2, the fruit shape of *C. reticulata* is primarily spherical. Among the different populations, the pericarp color is mainly of the orange-yellow (red) series (89.900%), with a minority being yellow-green color (11.100%). The seed shape is predominantly hemispherical (65.556%) and conical (30.556%), while spherical (2.778%) and reniform-like (1.111%) shapes are less common. The seed coat color is also mainly brown (96.667%), with a very small proportion being tan (3.333%) (Figure 3). For the different cultivars, the pericarp color is primarily of the orange-yellow (red) series. The seed shape is predominantly hemispherical (71.500%), with some being conical (15.667%) and spherical (12.333%); reniform-like shapes are rare (0.500%). The seed coat color is primarily brown (98.167%), with an extremely small proportion being tan (1.833%).

### 2.4. Analysis of Diversity in Fruit Quantitative Traits Among Different Populations and Cultivated Varieties

The F-test was used to analyze the differences among different populations and cultivars of *C. reticulata*. The results indicated that all nine phenotypic traits of the fruits exhibited highly significant differences among the nine populations and 30 cultivars (Table 3 and Table 4). The coefficient of variation (*CV*) for the nine phenotypic traits in different populations ranged from 17.209% to 60.803%, with an average of 31.655%. The traits ranked in descending order of *CV* were: SW (60.803%) > FW (49.536%) > SN (43.883%) > PT (32.642%) > FH (22.685%) > ON (21.835%) > SH (18.377%) > SL (17.924%) > FL (17.209%).

In contrast, the *CV* for the nine phenotypic traits in different cultivars ranged from 13.951% to 72.911%, with an average of 35.290%. The ranking was: SN (72.911%) > SW (67.243%) > FW (50.737%) > ON (35.271%) > PT (25.417%) > FH (18.614%) > FL (18.581%) > SL (14.883%) > SH (13.951%). The results demonstrate that significant variation in fruit phenotypic traits exists both among different populations and cultivars of *C. reticulata*. Among the populations, FL showed relative stability, whereas SW exhibited the most pronounced variation. In comparison, SH was relatively stable among cultivars, while SN displayed the greatest degree of variation.

Furthermore, the diversity indices for the nine phenotypic traits of *C. reticulata* fruits from different populations ranged from 1.273 to 1.735. Among these, FW and FL exhibited the highest diversity, both with an index of 1.735. FH and PT followed, each with a diversity index of 1.677. In contrast, SW showed the lowest diversity (1.273), while the ON and SN also had relatively low diversity indices of 1.279 and 1.311, respectively. For the cultivars, the diversity indices for the nine phenotypic traits ranged from 1.439 to 1.868. Specifically, FH displayed the highest diversity, with an index of 1.868. PT and FL followed closely, with diversity indices of 1.754 and 1.710, respectively. In comparison, SN showed the lowest diversity, with an index of only 1.439, while SW and SL also had relatively low diversity indices of 1.500 and 1.495, respectively.

**Figure 4:**
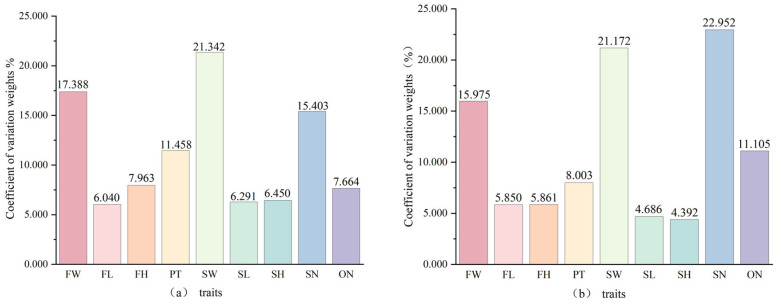
The weights of the coefficient of variation of nine phenotypic traits in different populations and fruits of different varieties of *C. reticulata*. (**a**): nine wild populations of *C. reticulata*. (**b**): 30 cultivars of *C. reticulata*. FW: Fruit Weight; FL: Fruit Transverse Diameter; FH: Fruit Longitudinal Diameter; PT: Peel Thickness; SW: Seed Fresh Weight; SL: Seed Transverse Diameter; SH: Seed Longitudinal Diameter; SN: Seed Number; ON: The Number of Locules.

The results of the weight calculation using the coefficient of variation method are shown in Figure 4. Regarding the coefficient of variation weights for the nine fruit phenotypic traits, those for the nine populations of *C. reticulata* different and ranked in descending order as follows: SW (21.342%) > FW (17.388%) > SN (15.403%) > PT (11.458%) > FH (7.963%) > ON (7.664%) > SH (6.450%) > SL (6.291%) > FL (6.040%). The maximum indicator weight was SW (21.342%), while the minimum was FL (6.040%). For the 30 cultivars, the coefficient of variation weights ranked in descending order as: SN (22.956%) > SW (21.172%) > FW (15.975%) > ON (11.105%) > PT (8.003%) > FH (5.861%) > FL (5.850%) > SL (4.686%) > SH (4.392%). Here, the maximum indicator weight was SN (22.956%), and the minimum was SH (4.392%). Among the different populations, SW, FW, and SN were also the traits that had a greater influence on fruit phenotypic traits. The remaining traits had a relatively smaller impact on *C. reticulata*, though they still exerted a certain degree of influence on the fruit phenotypes. Among the 30 cultivars, the variability in SN, SW, and FW had a more significant influence among the nine fruit phenotypic traits.

### 2.5. Correlation Analysis of Fruit Phenotypic Traits Among Different Populations and Cultivated Varieties

**Figure 5:**
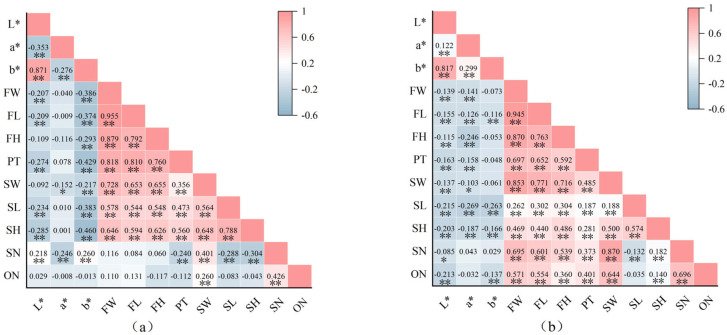
Correlation of *C. reticulata* fruit traits. (**a**): nine populations of *C. reticulata*. (**b**): 30 cultivars of *C. reticulata*. FW: Fruit Weight; FL: Fruit Transverse Diameter; FH: Fruit Longitudinal Diameter; PT: Peel Thickness; SW: Seed Fresh Weight; SL: Seed Transverse Diameter; SH: Seed Longitudinal Diameter; SN: Seed Number; ON: The Number of Locules. “*” means significant at 0.05 level, “**” means significant at 0.01.

The results of the correlation analysis (Figure 5) indicate that there are highly significant correlations (*p* < 0.05) among all fruit phenotypic traits. For the nine populations, the fruit L* value exhibited a highly significant negative correlation with the a* value, a highly significant positive correlation with the b* value (r = 0.871), and the a* value showed a highly significant negative correlation with the b* value. Furthermore, the L*, a*, and b* values either had highly significant negative correlations or showed no significant correlation with the nine quantitative phenotypic traits of the fruits. For the thirty cultivars, the fruit L* value showed highly significant positive correlations with both the a* and b* values, and the a* value also exhibited a highly significant positive correlation with the b* value. However, the L*, a*, and b* values displayed highly significant negative correlations or even no significant correlation with the nine quantitative phenotypic traits of the fruit.

Among the nine populations, FW maintained highly significant positive correlations with multiple traits, including FL (r = 0.955), FH (r = 0.879), PT (r = 0.818), SW, SL, and SH. However, no significant associations were found between FW, FL, and FH, and either SN or ON. PT, SL, and SH showed highly significant negative correlations with SN, while SW showed a highly significant positive correlation with SN (r = 0.870). Regarding ON, apart from highly significant positive correlations with SW and SN, no significant correlations were observed with other traits. Among the 30 cultivars, all nine quantitative traits of the fruits showed highly significant positive correlations with each other. Notably, FW showed particularly strong correlations with FL (r = 0.945), FH (r = 0.870), and SW (r = 0.853). In contrast, SH demonstrated a highly significant negative correlation with SN but showed no significant correlation with ON. These findings are of certain importance for understanding the fruit characteristics and their genetic basis in *C. reticulata*.

### 2.6. Principal Component Analysis of Fruit Phenotypic Traits Among Different Populations and Cultivated Varieties

The results of the principal component analysis (Table S3) are as follows: Based on the criterion of eigenvalues greater than one, two principal components were extracted. The cumulative contribution rate reached 76.064% for the populations and 73.570% for the cultivars, indicating that these two principal components captured most of the information from the nine quantitative phenotypic traits for both the populations and cultivars. Among the different populations, the first principal component had the largest eigenvalue of 4.948. In its corresponding eigenvector, FW had the largest absolute value (0.944), contributing 54.979% to the variance. The second principal component had an eigenvalue of 1.898, with SN showing the largest absolute value (0.912) in its eigenvector, bringing the cumulative contribution rate to 76.064%. Similarly, for the thirty cultivars, the first principal component had the largest eigenvalue of 5.047, and FW had the highest absolute value (0.962) in its eigenvector, contributing 56.077%. The second principal component had an eigenvalue of 1.574, with SN exhibiting the largest absolute value (0.815) in its eigenvector, resulting in a cumulative contribution rate of 73.570%. The comparative results indicate that these two principal components collectively captured and explained most of the variation in the phenotypic traits.

**Figure 6:**
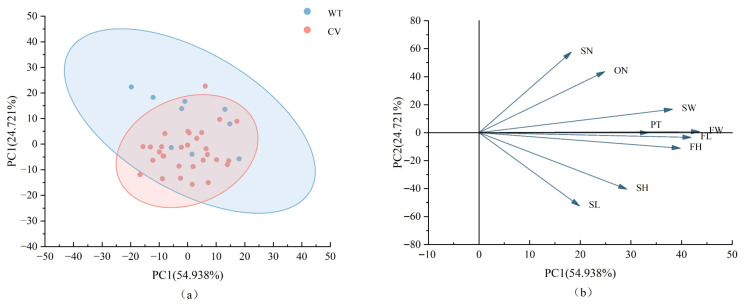
Principal component analysis of different populations and cultivars of *C. reticulata*: (**a**) PCA score plot, (**b**) PCA loading plot. WT: Different populations, CV: Cultivated varieties.

Furthermore, the PCA biplot for different populations and cultivars (Figure 6) reveals that populations are more widely dispersed, exhibiting richer phenotypic variation, while cultivars are more clustered, showing greater trait uniformity. The two groups demonstrate significant differentiation along the PC1 and PC2 axes. The loading plot indicates that the positive direction of PC1 is primarily driven by fruit size-related traits (FW, FL, FH, etc.), while the positive direction of PC2 is associated with seed number and locule number (SN, ON), and the negative direction is linked to seed size (SL, SH).

### 2.7. Comprehensive Evaluation of Fruit Phenotypic Traits in Different Populations and Cultivated Varieties

Through principal component analysis, the score coefficients for the nine quantitative phenotypic traits of fruits from different populations and cultivated varieties were obtained (Table 5). Furthermore, a linear combination model based on these two principal components as parameters was constructed. Using this model, the comprehensive coefficients for the populations and cultivars across the two principal components were calculated separately (Table 6 and Table 7). To further evaluate the germplasm resources of *C. reticulata*, this study established comprehensive evaluation models: for populations, F = (54.979F_1_ + 21.086F_2_)/76.065; for cultivars, F = (56.077F_1_ + 17.493F_2_)/73.570. These models integrate the contribution rates of each principal component for the systematic assessment of the overall performance of different populations and cultivars. The calculations from this model objectively reflect the fruit trait characteristics of different populations and cultivars, providing a scientific basis for germplasm resource evaluation and the selection of superior cultivars. In the comprehensive evaluation of the nine populations, the top three were Fengqing I (1.624), Hemu Village I (1.567), and Zixi Mountain II (1.459). In the comprehensive evaluation of the thirty cultivars, the top five were ‘Lichan’ (2.410), ‘Shizitou’ (2.148), ‘Kunming Chun’ (2.070), ‘Fengshan Cha’ (1.544), and ‘Lifang’ (1.404).

### 2.8. Cluster Analysis of Fruit Phenotypic Traits

**Figure 7:**
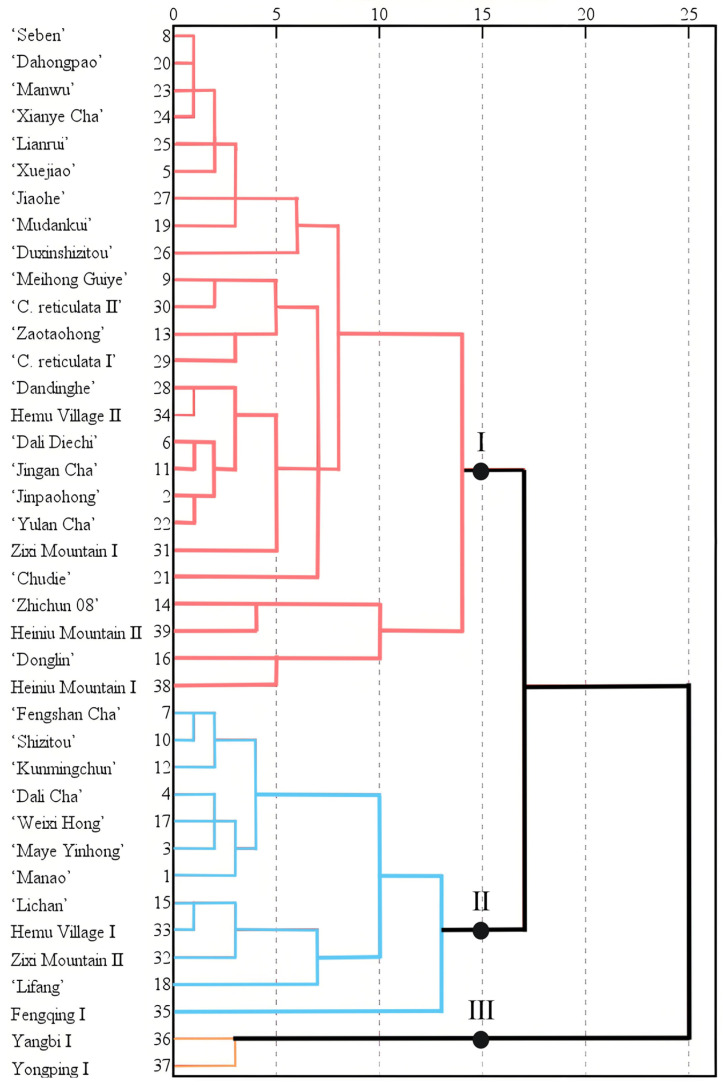
Cluster analysis of fruit phenotypic traits of different populations and cultivars of *C. reticulata*.

**Figure 8:**
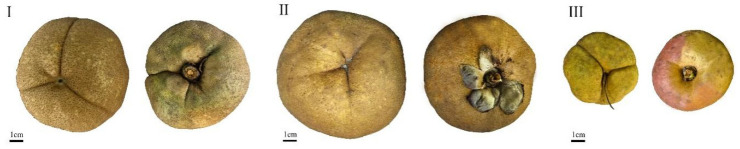
Examples of typical fruits in the three major groups of *C. reticulata*. Class I: *C. reticulata* ‘JinpaoHong’, Class II: *C. reticulata* ‘WeixiHong’, Class III: Yangbi wild *C. reticulata*.

Cluster analysis was performed on the nine quantitative phenotypic traits of fruits from nine populations and thirty cultivars of *C. reticulata*. At a Euclidean distance of 15.0, the resources of *C. reticulata* from both the nine populations and the thirty cultivars were divided into three major clusters (Figure 7 and Figure 8). The average coefficients of variation for these three clusters were 34.115%, 26.812%, and 18.621%, respectively, indicating rich genetic variation in the fruit phenotypes among these major groups (Table S4).

Cluster I comprises four populations: Hemu Village II, Zixi Mountain I, Heiniu Mountain I, and Heiniu Mountain II, along with 21 cultivars including ‘Seben’, ‘Dahongpao’, ‘Manwu’, ‘Lianrui’, ‘Xuejiao’, and ‘Jinpaohong’ (Figure 8). Compared to Clusters II and III (Table S4), this cluster exhibits intermediate mean values for FW, FL, FH, PT, SW, SL, and SH. In contrast, the mean values for SN and ON are relatively low. This cluster has the largest average coefficient of variation at 34.115%, with SN showing the highest coefficient of variation (74.917%) and SH the lowest (14.216%). These results indicate that the fruit morphological characteristics of *C. reticulata* in this cluster generally demonstrate an intermediate level of expression.

Cluster II comprises three populations: Hemu Village I, Zixi Mountain II (Figure 8), and Fengqing I, along with nine cultivars including ‘Fengshan Cha’, ‘Shizitou’, ‘Kunming Chun’, ‘Dali Cha’, ‘Weixi Hong’, and ‘Maye Yinhong’. Compared to Clusters I and III (Table S4), this cluster exhibits the highest mean values for FW, FL, FH, PT, SW, SL, SH, and ON, while the mean SN is at an intermediate level. The average coefficient of variation for this cluster is 26.812%, which is at an intermediate level among the clusters. Within this group, SN shows the highest coefficient of variation (57.879%), and SH the lowest (10.456%). These results indicate that the fruits and seeds of *C. reticulata* in this cluster are relatively large.

Cluster III comprises only two populations of *C. reticulata*: Yangbi I (Figure 8) and Yongping I. Compared to Clusters I and II (Table S4), this cluster exhibits relatively smaller mean values for FW, FL, FH, PT, SW, SL, and SH. In contrast, the mean SN is at its maximum, while the mean ON is at an intermediate level. The average coefficient of variation for this cluster is the smallest at 18.621%, with fruit weight showing the highest coefficient of variation (32.385%) and SH the lowest (9.158%). These results indicate that the fruits and seeds of *C. reticulata* in this cluster are relatively small, yet the traits demonstrate higher stability.

### 2.9. Boxplot Analysis of Fruit Phenotypic Traits in Different Populations and Cultivated Varieties

**Figure 9:**
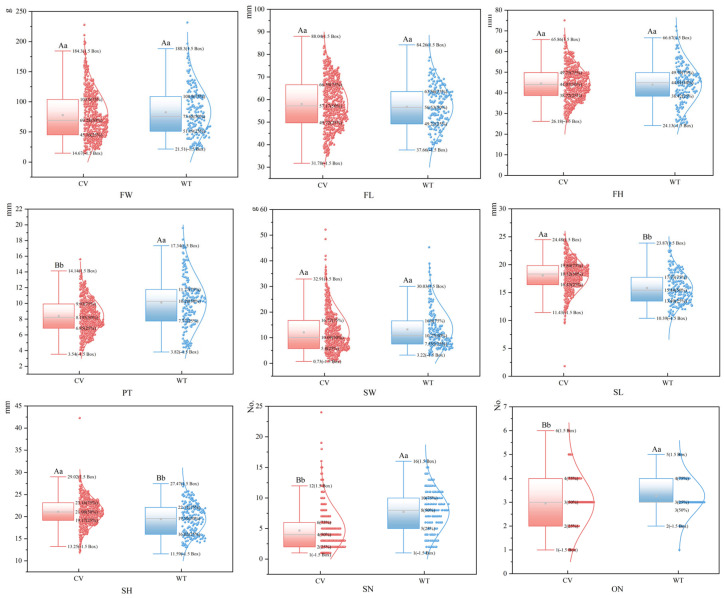
Box plots of fruit phenotypic traits of different populations and cultivars of *C. reticulata*. CV: Cultivated variety; WT: Different populations. The middle area (the box) of each boxplot contains 50% of the individuals, and the area between the upper and lower horizontal lines contains 90% of the individual means in the form of small squares in the box. Different lowercase letters indicated significant differences in the mean values of phenotypic traits in fruits (*p* < 0.05), and different uppercase letters indicated extremely significant differences in the mean values of fruit phenotypic traits (*p* < 0.01). The same letters indicate no significant differences.

The nine quantitative phenotypic traits of *C. reticulata* fruits were visualized using box plots, reflecting the number, mass, and dimensions of the fruits and seeds. A comparative analysis was conducted between different populations and cultivars. The results indicate that, except for FL, SL, SH, and ON, the distribution of fruit traits in cultivars generally shifted downward compared to that in different populations. The specific distributions of all phenotypic traits are presented as follows (Figure 9).

In terms of quantity, the distribution of SN in different populations of *C. reticulata* is broader than that in cultivars. Conversely, the distribution of ON in cultivars spans a wider range, completely encompassing the distribution observed in different populations. Additionally, the proportion of outliers for SN among cultivars is 4.000%. Regarding mass and dimensions, the distributions of FH, PT, SL, and SH are broader in different populations compared to cultivars. In contrast, the distributions of FL, SW, and FW are more extensive in cultivars than in different populations of *C. reticulata*. Additionally, the distributions of SL and SH are more concentrated (denser) in cultivars. For the traits of FW, FH, and PT, the proportion of outliers in different populations was 1.111%, respectively. In cultivars, the proportions of outliers for these three traits were lower, at 0.833%, 0.500%, and 0.167%, respectively. Among different populations, only SW exhibited outliers, with a proportion of 4.444%. For cultivars, the proportions of outliers for SL, SH, and SW were 1.000%, 0.500%, and 2.000%, respectively.

### 2.10. Frequency Distribution Function Analysis of Fruit Phenotypic Trait Variation in Different Populations and Cultivated Varieties

**Figure 10:**
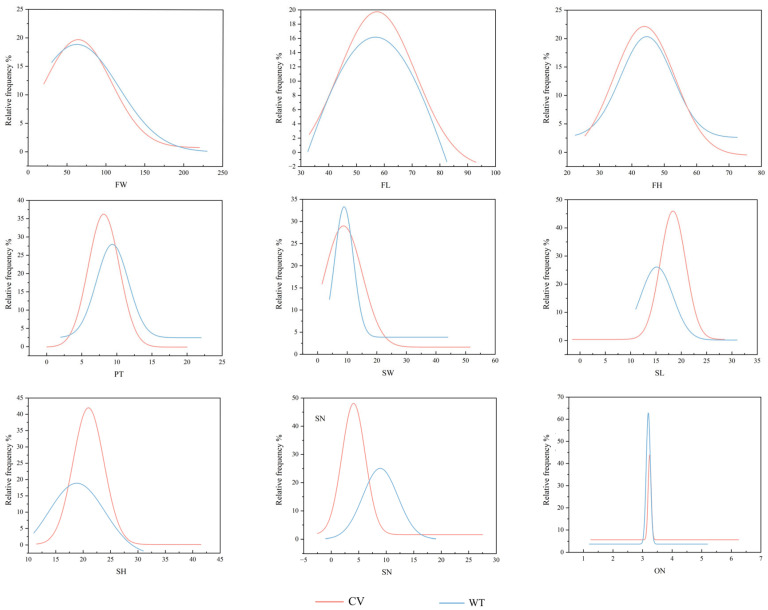
Frequency distribution of phenotypic traits in different populations and cultivars of *C. reticulata*. CV: Cultivated variety; WT: Different populations FW: Fruit Weight; FL: Fruit Transverse Diameter; FH: Fruit Longitudinal Diameter; PT: Peel Thickness; SW: Seed Fresh Weight; SL: Seed Transverse Diameter; SH: Seed Longitudinal Diameter; SN: Seed Number; ON: The Number of Locules.

To further analyze the variation in fruit phenotypic traits among different populations and cultivars of *C. reticulata*, frequency distribution functions were fitted for the nine quantitative phenotypic traits of the fruits (Figure 10). All fruit organ phenotypic traits followed a normal distribution (R^2^ = 0.673–0.999) (Table S5). A comparison of the frequency distribution functions between the two categories, different populations, and cultivars revealed that, except for the power distribution functions of FW, FH, and ON, which were relatively concentrated, the power function distributions of the remaining six traits were more dispersed. This further indicates substantial variability in the fruit phenotypic traits between populations and cultivars.

Regarding mass traits, SW exhibited both vertical and horizontal shifts in its distribution, indicating greater variability in this trait. In contrast, the power distribution function of FW showed no significant shift, suggesting relatively lower variability. For size traits, FL, PT, and SH displayed vertical and horizontal shifts in their distributions, indicating greater variability in these traits. Conversely, the fruit shape index (FL to FH ratio) showed no significant horizontal shift, and the power distribution function of FH also lacked significant shifts, suggesting lower variability in these two traits. In terms of number traits, the normal distribution function of SN in populations was right-skewed compared to that in cultivars, indicating greater variability in populations. On the other hand, the power distribution function of ON showed no significant shift, suggesting lower variability in this trait.

## 3. Discussion

Phenotypic diversity, as a direct manifestation of genetic diversity, serves as a crucial entry point for analyzing the genetic structure, adaptation mechanisms, and evolutionary potential of species [29]. Methods such as correlation, clustering, and principal component analysis can systematically reveal the genetic characteristics and variation patterns of germplasm resources [30]. In cross-pollinated and often self-incompatible species like oil-tea camellia, the genetic origin of different traits significantly influences their variation patterns. Maternal tissue traits, such as pericarp characteristics, fully reflect the maternal genetic background, whereas seed traits integrate the nuclear genetic contributions from both parents, theoretically possessing greater potential for genetic variation [31,32]. Therefore, an in-depth exploration of the phenotypic characteristics of *C. reticulata* fruits will facilitate the efficient screening of germplasm resources with superior traits.

This study conducted an integrated analysis of 13 fruit phenotypic traits from nine wild populations and 30 cultivated varieties of *C. reticulata*. Both qualitative traits (fruit shape, pericarp color, seed shape, seed coat color) and quantitative traits (fruit size, seed size, etc.) were incorporated into a unified evaluation framework to comprehensively present the genetic variation pattern of fruit phenotypes. Regarding qualitative traits, the fruit shape was predominantly spherical. The pericarp color primarily fell within an orange-yellow (red) series (found in eight populations and all 30 cultivars), with the sole exception of the Heiniu Mountain I population, which exhibits a yellow-green series. This yellow-green series displayed distinct distribution characteristics in terms of its a* value, making it a potential key indicator for cultivar identification. Considering the influence of the environment on phenotypes, this color differentiation may stem from geographical adaptation or genetic divergence among different populations [33]. It also reflects the genetic conservation and differentiation trend of the pericarp, as a maternal trait, between wild and cultivated groups. Seed shape was mainly hemispherical (65.6%) and conical (30.6%) within the wild populations, while hemispherical seeds were even more predominant in cultivated varieties (71.5%). Seed coat color was overwhelmingly brown in both groups (96.7% in populations, 98.2% in varieties). Diversity analysis of quantitative traits revealed significant variation in the phenotypic traits of *C. reticulata*. The Shannon diversity index (*H′*) ranged from 1.270 to 1.870. Compared to existing studies, this range is lower than the average diversity index (*H′* = 1.8850) reported for seed and fruit traits of *C. chekiangoleosa* [18], and significantly lower than the diversity levels reported for *C. vietnamensis* (*H′* = 2.7499) [34] and for *C. meiocarpa* (*H′* = 2.8160) [35]. This discrepancy may reflect inherent differences in genetic diversity among species and simultaneously suggests considerable potential for enhancing the conservation and utilization of genetic resources for *C. reticulata*.

To analyze the effects of natural evolution and artificial selection on phenotypic traits, populations and cultivated varieties were processed separately to systematically evaluate the diversity composition and breeding potential of *C. reticulata*. The coefficient of variation, serving as a key metric for measuring trait dispersion, can intuitively reflect the fluctuation characteristics of phenotypic traits [36]. Analysis of the coefficient of variation revealed extensive variation across the nine quantitative traits of *C. reticulata* (13.950% ~ 72.910%). The average coefficient of variation for cultivated varieties (35.290%) was slightly higher than that for populations (31.655%), yet both were significantly lower than that reported for wild *C. oleifera* (88.630%) [37]. Among populations, SW exhibited the most pronounced variation (60.800%), consistent with findings reported by He Yimin, Jin Gaozhong et al. [38,39]. Among cultivated varieties, SN showed the highest coefficient of variation (72.910%), aligning with results from studies on *C. meiocarpa* in Guizhou [40]. Furthermore, a negative correlation was observed between diversity indices and coefficients of variation across different populations and varieties of *C. reticulata*. This pattern is consistent with findings in plants such as *Nymphaea* [41] and *Machilus gamblei* [42], suggesting that this phenomenon may represent a common characteristic of phenotypic diversity in plants.

Correlation analysis of fruit phenotypic traits provides an important theoretical foundation for cultivar improvement and biotechnological research [43]. The correlation analysis revealed highly significant positive correlations among most traits in *C. reticulata*. FW showed highly significant positive correlations with key morphological indicators. In populations, FW was highly correlated with FL (r = 0.955), FH (r = 0.879), and PT (r = 0.818). In cultivated varieties, besides strong correlations with FL (r = 0.945) and FH (r = 0.870), it also exhibited a strong correlation with SW (r = 0.853). Notably, in both populations and varieties, all metrics reflecting fruit size and seed size demonstrated highly significant positive correlations. This finding aligns with results from studies on *Olea europaea* [44], providing new evidence for understanding the co-evolutionary mechanisms of fruit and seed traits in woody oil plants.

Principal component analysis (PCA) aims to reduce the dimensionality of morphological trait information while minimizing the loss of original data, thereby simplifying trait classification and focusing on core elements [45]. The PCA revealed that PC1 primarily represents variation in traits related to fruit size, while PC2 mainly reflects variation in reproductive allocation traits associated with seed number. Together, they account for the majority of phenotypic differences among the groups. This finding further supports prioritizing fruit weight and seed number as key trait dimensions in subsequent genetic analysis and breeding efforts. Cluster analysis is capable of revealing the genetic structure and variation patterns of plant germplasm resources, providing crucial theoretical support for plant breeding [46]. The cluster analysis further classified the materials into three groups: Group I exhibits superior comprehensive traits and is suitable as high-quality parental material; Group II demonstrates prominent ornamental value and adaptability, making it suitable for specialized breeding; Group III shows stable phenotypes and is applicable for specific research or applications.

Comprehensive evaluation of plant germplasm resources is a core component of breeding research and holds significant guiding value for cultivar selection [47]. The trait-based comprehensive evaluation indicated that the cultivated variety ‘Lichan’ achieved the highest score (F = 2.410), significantly outperforming the wild population Fengqing I (F = 1.624), thus positioning it as a core parental line for genetic improvement. Boxplot analysis identified outlier individuals for specific traits, whose variation may stem from gene mutations, specific ecological environmental factors, genetic recombination, or other genetic mechanisms. These findings provide a reference for selecting new germplasm. Frequency distribution analysis demonstrated that all traits across different populations and varieties conformed to a normal distribution (R^2^ = 0.673~0.999), further confirming the continuity and analyzability of phenotypic variation. Through a systematic analysis of phenotypic diversity in wild and cultivated populations of *C. reticulata*, this study clarified their variation characteristics, key traits, and inter-trait correlations. The findings provide a theoretical foundation for the subsequent utilization of germplasm resources, genetic improvement, and application within the tea oil industry.

## 4. Materials and Methods

### 4.1. Materials

#### 4.1.1. Experimental Site Overview

The fruit sampling area for this study spans a wide geographical range with notable altitudinal gradients, covering three climatic zones: mid-subtropical, north-subtropical, and warm temperate. The overall region falls within the subtropical plateau monsoon climate zone, exhibiting distinct vertical climatic differentiation. The fruits from the nine populations were named according to their collection sites (Table 8). The 30 cultivars were collected from the following locations: Zhengyang Camellia Garden Landscape Co., Ltd. in Yiliang County, Kunming City (103°23′ E, 24°88′ N, altitude 1950 m); Zixi Mountain, Chuxiong City (101°24′ E, 25°00′ N, altitude 2386 m); Dongjia Garden, Huaning County (102°56′ E, 24°12′ N, altitude 1633 m); Laifeng Mountain, Tengchong City (98°29′ E, 25°10′ N, altitude 1736 m); Xiaojia Garden in Tengchong City (98°28′ E, 25°50′ N, altitude 1871 m); and Shidong Temple, Fengqing County (100°20′ E, 24°29′ N, altitude 2223 m). Among these, the samples collected from Xiaojiayuan, Tengchong City, were respectively named as ‘*C. reticulata* I’ and ‘*C. reticulata* II’ (Table 9).

#### 4.1.2. Plant Materials

This study utilized fruits of *C. reticulata*, collected from nine natural populations and 30 cultivated varieties, as experimental materials. All fruit samples were obtained from vigorously growing plants and were harvested between September and mid-October 2024. For each wild population, 30 maternal trees were randomly selected, and 12 mature fruits were randomly collected from each tree. The fruits collected from these 30 maternal trees (12 × 30 = 360) were thoroughly mixed, after which 20 fruits were randomly chosen as experimental materials for that population. For each cultivated variety, no fewer than 10 representative individual plants were selected as maternal sources for sampling, and a total of 20 fruits were randomly collected to ensure adequate representation of maternal genetic backgrounds.

### 4.2. Fruit Phenotypic Trait Parameters

In this study, with references to the classification of camellia cultivars in China, testing guidelines, and known cultivar databases [48], the phenotypic traits of fruits from *C. reticulata* were classified into two categories: qualitative traits and quantitative traits. Qualitative traits are typically controlled by single genes and exhibit discrete states, while quantitative traits are regulated by multiple genes and show continuous variation [49]. This classification provides a theoretical foundation and methodological basis for subsequent targeted diversity analysis.

**Figure 11:**
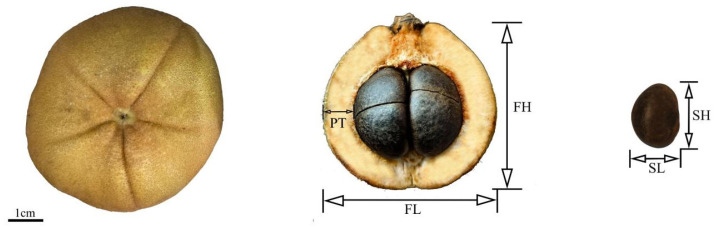
Schematic diagram of the determination of phenotypic traits in fruits. Taking *C. reticulata* ‘Dali Diechi’ as an example: FL: fruit transverse diameter; FH: longitudinal diameter of fruit; PT: peel thickness; SL: seed transverse diameter; SH: seed longitudinal diameter.

Within 24 h of collection, the selected fruits were measured for shape, size, and weight. After the fruits had naturally split open, measurements were taken for pericarp thickness, locule number, and seed shape, size, quantity, and weight of the seeds (Figure 11). Furthermore, the statistical indicators were divided into two categories:

Category I comprises quantitative traits, including a total of nine indicators: fruit transverse and longitudinal diameter (mm), fruit weight (g), pericarp thickness (mm), number of fruit locules, seed transverse and longitudinal diameter (mm), seed weight (g), and seed count (Table 10).

Category II comprises qualitative traits, including four indicators: pericarp color, seed coat color, fruit shape, and seed shape. The classification and statistics for Category II indicators were performed according to NY/T 2943-2016 [50], the descriptor standard for tea plant germplasm resources, with the classification criteria detailed in Table 11. The pericarp color was graded using cluster analysis, while seed coat color was primarily assessed through visual inspection.

**Table 11:** The grading criteria for quantitative traits.

Quantitative Trait Indicators	Grading Criteria				
	1	2	3	4	5
Peel color	Orange (Red) color	Yellow-green color			
Fruit shape	Spherical	Kidney-shaped	Triangular	Square	lum-blossom-shaped
Seed shape	Spherical	Hemispherical	Conical	Kidney-shaped	Irregular
Seed coat color	Brown	Sepia	Dark brown		

### 4.3. Pericarp Color Parameters

The pericarp color differences in nine populations and thirty cultivars of *C. reticulata* were analyzed using a combination of traditional visual assessment and colorimetry based on the standard CIELab color system (3nh, Precision Colorimeter NR 110, Shenzhen ThreeNH Technology Co., Ltd., Shenzhen, China). Twenty biological replicates were set for each sample. The measured parameters included Lightness (L*) and two chromaticity components, a* and b*. The values of chroma (C*) and hue angle (*h°*) were calculated according to the formulas [51]:

C* = (a*2 + b*2)1/2,

*h°* = arctan (b*/a*)

### 4.4. Data Analysis

Statistical analysis of the quantitative traits collected from the nine populations and thirty cultivars of *C. reticulata* was conducted using Excel 2020. Frequency distribution and genetic diversity index (*H′*) were evaluated for both qualitative and quantitative traits. Further statistical characteristics of the quantitative traits were calculated, including means, maximum (Max), minimum (Min), standard deviation (S), range, and coefficient of variation (*CV*).

For the quantitative traits of the test materials, all data were divided into six grades based on the following criteria: Grade 1 [Xi < (X‾ − S)] to Grade 6 [Xi > (X‾ + S)], with each grade spanning 0.5S. Here, X‾ represents the mean, S denotes the standard deviation, and Xi corresponds to the data in the i-th grade [52]. The relative frequency (Pi) of each grade was used to calculate the Shannon–Weiner diversity index (*H*′), using the formula:

*H′* = −∑ (Pi × lnPi),

where Pi represents the percentage of materials in the i-th grade relative to the total number of materials for a given trait [53].

Using SPSS 23.0 software, hierarchical clustering analysis was performed based on Euclidean distance to analyze the pericarp color parameters (L*, a*, and b*) and the diversity of fruit phenotypic traits for the nine populations and thirty cultivars of *C. reticulata*. A cluster diagram was generated accordingly. Data standardization was achieved using the Kaiser method, and principal component analysis (PCA) was conducted, with components extracted based on eigenvalues of no less than 1.00 [54].

Boxplots were constructed as follows: the box covers 50% of the observation range, the region between the upper and lower whiskers covers 90% of the observation range, and values outside the whiskers were considered outliers. The focus of this study was on the box and the mean. The mean values were used for Duncan’s multiple comparison test to determine significant differences among different populations and cultivars of *C. reticulata* (*p* < 0.05 and *p* < 0.01 were considered significant and highly significant, respectively). Outliers were analyzed to reflect extreme variations [55], which is crucial for germplasm innovation in *C. reticulata*. Frequency distribution function analysis was performed using Excel 2020, Origin 2024, and SPSS 23.0 software to fit frequency distribution functions for the nine quantitative phenotypic traits of the fruits.

## 5. Conclusions

This study systematically analyzed 13 fruit phenotypic traits across nine wild populations and 30 cultivated varieties of *C. reticulata*. The results revealed significant phenotypic differentiation between the two groups, with fruit size and seed number identified as key traits driving this variation. These finding provides an important phenotypic basis for selecting high-yield and high-oil varieties. A negative correlation was observed between the phenotypic diversity index and the coefficient of variation within populations, which may be related to the genetic structure associated with cross-pollination and self-incompatibility systems. In the comprehensive evaluation, the cultivar ‘Lichan’ demonstrated the best performance, making it a preferred parent for genetic improvement. The results systematically elucidate the patterns of trait variation and genetic characteristics of this species, emphasizing the need in breeding to balance the stability of maternal traits with the potential heterosis of biparental traits. This study provides both theoretical and practical foundations for germplasm resource evaluation, parental selection, and the breeding of high-yield and high-oil varieties.

## Figures and Tables

**Table 1:** Diversity analysis of qualitative traits in different populations of *Camellia reticulata*.

Quality Traits	Frequency of Classific/%				
	1	2	3	4	5
Peel color	88.900	11.100			
Fruit shape	100.000	0.000	0.000	0.000	0.000
Seed shape	2.778	65.556	30.556	1.111	0.00
Seed coat color	0.000	3.333	96.667		

**Table 2:** Diversity analysis of qualitative traits of 30 cultivars of *C. reticulata*.

Quality Traits	Frequency of Classific/%				
	1	2	3	4	5
Peel color	100.000	0.000			
Fruit shape	100.000	0.000	0.000	0.000	0.000
Seed shape	12.333	71.500	15.667	0.500	0.000
Seed coat color	0.000	1.833	98.167		

**Table 3:** Analysis of diversity in fruit quantitative phenotypic traits from nine populations of *C. reticulata*.

Traits	Mean	Min	Max	Range	*CV*/%	F	*H′*
FW	82.516 ± 40.875	21.510	231.570	210.060	49.536	73.117 **	1.735
FL	56.738 ± 9.764	37.860	84.260	46.400	17.209	74.582 **	1.735
FH	43.959 ± 9.972	24.130	72.200	48.070	22.685	113.206 **	1.677
PT	10.100 ± 3.297	3.820	19.610	15.790	32.642	140.298 **	1.677
SW	13.195 ± 8.023	3.220	45.260	42.040	60.803	47.853 **	1.273
SL	15.819 ± 2.835	10.390	23.870	13.480	17.924	22.684 **	1.523
SH	19.438 ± 3.572	11.590	27.470	15.880	18.377	47.616 **	1.427
SN	7.700 ± 3.379	1.000	16.000	15.000	43.883	28.404 **	1.311
ON	3.270 ± 0.714	1.000	5.000	4.000	21.835	14.460 **	1.279

‘**’ means significant at the 0.01 level.

**Table 4:** Diversity analysis of fruit quantitative phenotypic traits of 30 Cultivars of *C. reticulata*.

Traits	Mean	Min	Max	Range	*CV*/%	F	*H′*
FW	77.771 ± 39.459	14.670	227.660	212.990	50.737	17.513 **	1.693
FL	58.028 ± 10.782	31.780	88.040	56.260	18.581	23.827 **	1.710
FH	44.474 ± 8.278	26.180	75.110	48.930	18.614	26.523 **	1.868
PT	8.391 ± 2.133	3.540	15.620	12.080	25.417	26.328 **	1.754
SW	12.069 ± 8.166	0.730	52.160	51.430	67.243	13.940 **	1.500
SL	18.057 ± 2.687	1.820	25.400	23.580	14.883	14.148 **	1.495
SH	21.114 ± 2.946	12.010	42.270	30.260	13.951	16.000 **	1.673
SN	4.670 ± 3.405	1.000	24.000	23.000	72.911	15.754 **	1.439
ON	2.950 ± 1.040	1.000	6.000	5.000	35.271	14.092 **	1.590

‘**’ means significant at the 0.01 level. FW: Fruit Weight; FL: Fruit Transverse Diameter; FH: Fruit Longitudinal Diameter; PT: Peel Thickness; SW: Seed Fresh Weight; SL: Seed Transverse Diameter; SH: Seed Longitudinal Diameter; SN: Seed Number; ON: The Number of Locules.

**Table 5:** Scoring coefficients of fruit traits of different populations and cultivars of *C. reticulata*.

Traits	Population Score Coefficient		Cultivar Score Coefficient	
	PC1	PC2	PC1	PC2
FW	0.185	0.104	0.173	0.081
FL	0.181	0.092	0.151	0.109
FH	0.186	−0.005	0.112	0.162
PT	0.176	−0.112	0.109	0.092
SW	0.130	0.267	0.203	−0.022
SL	0.155	−0.157	−0.147	0.499
SH	0.169	−0.123	−0.059	0.411
SN	−0.036	0.480	0.259	−0.250
ON	−0.018	0.391	0.224	−0.199

FW: Fruit Weight; FL: Fruit Transverse Diameter; FH: Fruit Longitudinal Diameter; PT: Peel Thickness; SW: Seed Fresh Weight; SL: Seed Transverse Diameter; SH: Seed Longitudinal Diameter; SN: Seed Number; ON: The Number of Locules.

**Table 6:** Comprehensive coefficients of fruit traits of nine populations of *C. reticulata*.

No.	Populations	Principal Component Values			Ranking
		F_1_	F_2_	F	
5	Fengqing I	3.511	−1.453	1.624	1
3	Hemu Village I	2.417	1.129	1.567	2
2	Zixi Mountain II	1.187	2.182	1.459	3
1	Zixi Mountain I	0.595	−1.931	−0.080	4
8	Heiniu Mountain I	−0.609	0.947	−0.135	5
9	Heiniu Mountain II	−0.585	−0.075	−0.338	6
4	Hemu Village II	−0.689	−1.649	−0.727	7
6	Yangbi I	−2.496	0.263	−1.317	8
7	Yongping I	−3.961	0.588	−2.054	9

**Table 7:** Comprehensive coefficients of fruit traits of 30 *C. reticulata* cultivars.

No.	Cultivars	Principal Component Values			Ranking
		F_1_	F_2_	F	
15	‘Lichan’	4.578	−0.898	2.410	1
10	‘Shizitou’	3.393	1.400	2.148	2
12	‘Kunmingchun’	3.293	1.447	2.070	3
7	‘Fengshan Cha’	2.372	1.224	1.544	4
18	‘Lifang’	3.096	−1.902	1.404	5
1	‘Manao’	1.470	2.460	1.254	6
17	‘Weixi Hong’	1.702	0.566	1.054	7
3	‘Maye Yinhong’	1.569	0.191	0.913	8
4	‘Dali Cha’	1.185	1.025	0.844	9
2	‘Jinpaohong’	1.444	−0.814	0.667	10
16	‘Donglin’	2.183	−3.676	0.581	11
9	‘Meihong Guiye’	0.141	2.403	0.500	12
30	*‘C. reticulata* II’	0.456	1.164	0.459	13
14	‘Zhichun 08’	0.942	−1.099	0.336	14
11	‘Jingan Cha’	0.105	−0.232	0.018	15
6	‘Dali Diechi’	0.323	−0.956	0.014	16
22	‘Yulan Cha’	0.289	−1.117	−0.033	17
13	‘Zaotaohong’	−0.794	1.576	−0.170	18
28	‘Dandinghe’	−0.429	−0.329	−0.298	19
21	‘Chudie’	−0.867	0.627	−0.377	20
29	*‘C. reticulata* I’	−2.317	1.316	−1.069	21
23	‘Manwu’	−2.007	−0.112	−1.145	22
8	‘Seben’	−2.122	−0.157	−1.217	23
19	‘Mudankui’	−1.705	−1.732	−1.259	24
20	‘Dahongpao’	−2.152	−0.740	−1.336	25
24	‘Xianye Cha’	−2.343	−0.365	−1.378	26
25	‘Lianrui’	−2.913	−0.064	−1.645	27
5	‘Xuejiao’	−3.082	−0.647	−1.842	28
26	‘Duxinshizitou’	−4.093	0.566	−2.196	29
27	‘Jiaohe’	−3.664	−1.126	−2.252	30

**Table 8:** The list of nine natural populations of *C. reticulata*.

No.	Name	Sampling Location	Latitude/m	Longitude (E)	Altitude (N)
1	Zixi Mountain I	Chuxiong Zixi Mountain	2172 m	101°23′ E	24°59′ N
2	Zixi Mountain II		2386 m	101°24′ E	25°00′ N
3	Hemu Village I	Hemu Village (Tengchong City)	1967 m	98°30′ E	25°10′ N
4	Hemu Village II		1959 m	98°30′ E	25°10′ N
5	Fengqing I	Fengqing County (Lincang City)	2073 m	100°20′ E	24°27′ N
6	Yangbi I	Yangbi County (Dali City)	2489 m	99°51′ E	25°38′ N
7	Yongping I	Shili Meihua Village (Yongping County)	2456 m	99°38′ E	25°30′ N
8	Heiniu Mountain I	Heiniu Mountain (Chuxiong City)	2338 m	101°50′ E	24°53′ N
9	Heiniu Mountain II		2350 m	101°50′ E	24°53′ N

**Table 9:** The thirty cultivars of *C. reticulata* used in this study.

No.	Cultivars	Sampling Location	No.	Cultivars	Sampling Location
1	‘Manao’	Zhengyang Camellia (Yiliang County)	16	‘Donglin’	Chuxiong Zixi Mountain
2	‘Jinpaohong’		17	‘Weixi Hong’	
3	‘Maye Yinhong’		18	‘Lifang’	
4	‘Dali Cha’		19	‘Mudankui’	
5	‘Xuejiao’		20	‘Dahongpao’	Dong’s Garden (Huaning County)
6	‘Dali Diechi’		21	‘Chudie’	
7	‘Fengshan Cha’		22	‘Yulan Cha’	Laifeng Mountain (Tengchong City)
8	‘Seben’		23	‘Manwu’	
9	‘Meihong Guiye’		24	‘Xianye Cha’	
10	‘Shizitou’		25	‘Lianrui’	
11	‘Jingan Cha’		26	‘Duxinshizitou’	
12	‘Kunmingchun’	Zhengyang Camellia (Yiliang County)	27	‘Jiaohe’	Laifeng Mountain (Tengchong City)
13	‘Zaotaohong’		28	‘Dandinghe’	Shidong Temple (Fengqing County)
14	‘Zhichun 08’	Chuxiong Zixi Mountain	29	‘*C. reticulata* I’	Xiaojiayuan Garden (Tengchong City)
15	‘Lichan’		30	‘*C. reticulata* II’	

**Table 10:** Nine quantitative traits of *C. reticulata* fruit and their codes.

Number	Codes	Trait Types	Unit
1	FW	Fruit Weight	g
2	FL	Fruit Transverse Diameter	mm
3	FH	Fruit Longitudinal Diameter	mm
4	PT	Pericarp thickness	mm
5	SW	Seed Weight	g
6	SL	Seed Transverse Diameter	mm
7	SH	Seed Longitudinal Diameter	mm
8	SN	Seed Number	count
9	ON	The Number of Locules	locules

## Data Availability

The original contributions presented in this study are included in the article/Supplementary Material. Further inquiries can be directed to the corresponding author.

## Supplementary Materials

The supporting information can be downloaded at: https://www.mdpi.com/article/10.3390/plants15050771/s1. Table S1. Differential analysis of phenotypic traits in *C. reticulata*. Table S2. Analysis of fruit color phenotypic differences between *C. reticulata* populations and its cultivars. Table S3. Principal component analysis of fruit phenotypic traits in different populations and cultivars of *C. reticulata*. Table S4. Analysis of various morphological traits of different populations and cultivars of *C. reticulata*. Table S5. Frequency distribution function of fruit phenotypic traits of different populations and cultivars of *C. reticulata*.
