# Supplementary material for: Diversity Analysis of Fruit Phenotypic Traits in Camellia reticulata
Source: Plants (Basel). 2026 Mar 3;15(5):771. doi: 10.3390/plants15050771 (PMC12986874; doi:10.3390/plants15050771)
Supplement: Supplementary file 1 [file plants-15-00771-s001.zip › plants-4104674-supplementary.pdf]

Table S1 Differential analysis of phenotypic traits in *C. reticulata*

No.	Cultivars	FW	FH	FL	PT	SW	SL	SH	SN	ON
1	'Manao'	100.474±40.770 defgh	66.212±9.016 bcd	46.621±5.889 fghi	9.742±1.900 fgh	13.225±8.080 efghij	21.192±1.780 a	24.247±1.117 a	3.057±2.089 klmno	2.750±0.910 ijkl
2	'Jinpaohong'	85.278±33.111 ghij	58.894±7.814 fghi	47.209±8.460 efg	8.933±1.604 hijk	17.280±7.436 cde	17.917±1.595 fghij	21.220±2.087 efghij	7.100±3.042 ef	3.600±0.598 cde
3	'Maye Yinhong'	98.164±23.403 efgh	66.888±6.355 bcd	45.762±3.193 ghij	11.161±1.479 cd	10.407±4.643 hijklmnop	18.418±1.961 defgh	21.609±2.131 efgh	4.250±1.251 ghijkl	3.700±0.733 cd
4	'Dali Cha'	85.968±39.808 fghij	62.789±9.799 def	47.893±6.404 efg	8.506±1.620 jklm	14.050±8.597 defghi	20.324±1.428 ab	22.303±2.895 cde	3.850±2.681 hijklm	3.600±0.940 cde
5	'Xuejiao'	34.527±11.694 qrs	43.302±4.683 qr	34.845±4.138 n	5.806±1.041 tu	7.128±3.405 mnopqrs	17.557±2.482 ghij	19.957±1.625 ijkl	3.700±1.218 hijklm	3.050±0.224 efghijk
6	'Dali Diechi'	74.402±38.143 ijklm	58.360±10.345 fghi	41.513±5.553 kl	9.195±1.535 ghij	11.644±7.349 ghijklm	17.927±1.889 fghij	20.109±2.309 hijkl	5.850±3.829 fg	3.800±1.056 cd
7	'Fengshan Cha'	102.635±42.946 defgh	67.086±9.210 bcd	47.944±6.440 efg	8.840±2.004 hijk	18.652±10.881 bc	20.616±1.851 ab	23.604±1.878 abc	5.400±3.169 fgh	3.400±1.142 cdefg
8	'Seben'	55.021±20.214 lmnop	53.049±6.582 ijklmn	41.952±5.445 kl	6.115±0.926 stu	6.743±3.388 nopqrs	17.242±2.253 ghijk	20.066±2.231 hijkl	2.800±1.765 klmno	2.700±0.470 jkl
9	'Meihong Guiye'	73.386±32.155 ijklm	55.580±7.520 hijkl	48.592±6.858 defg	8.358±1.728 jklmn	11.230±5.796 ghijklmn	20.621±1.425 ab	24.630±2.558 a	2.900±1.518 klmno	2.600±0.940 kl
10	'Shizitou'	118.298±26.557 bcd	70.693±5.135 ab	51.731±4.951 cd	10.548±0.970 def	18.842±5.219 bc	20.195±1.566 abc	24.245±1.230 a	5.100±1.744 ghij	3.500±0.827 cdef
11	'Jingan Cha'	75.656±36.650 ijk	57.034±8.946 ghijk	42.707±6.030 jk	8.620±1.839 ijklm	11.384±8.313 ghijklm	18.012±2.095 fghij	21.530±2.513 efghi	5.100±3.999 ghij	3.400±1.142 cdefg
12	'Kunmingchun'	133.454±39.237 ab	72.929±6.002 a	55.664±5.833 b	8.374±1.362 jklmn	17.674±6.663 cd	20.260±2.083 abc	23.451±1.641 abc	5.550±2.625 fgh	3.000±0.725 fghijk
13	'Zaotaohong'	74.915±35.147 ijkl	58.842±8.524 fghi	45.933±4.608 ghij	6.797±1.280 qrs	9.514±7.810 jklmnopq	19.664±1.030 bcde	22.062±1.406 cdef	3.200±2.931 jklmno	2.050±0.999 mn

Continued Table S1

No.	Cultivars	FW	FH	FL	PT	SW	SL	SH	SN	ON
14	'Zhichun 08'	104.195±22.891 cdefg	64.853±4.150 cde	49.917±5.224 def	11.933±1.598 bc	9.449±3.030 jklmnopqr	15.802±3.624 kl	18.137±2.335 no	4.100±1.294 fhijkl	2.900±0.718 ghijkl
15	'Lichan'	117.616±3.051 bcde	66.977±6.776 bcd	50.146±6.830 def	10.945±1.589 de	28.204±11.203 a	17.828±3.092 fghij	23.934±2.474 ab	9.950±4.673 bc	3.950±0.605 bc
16	'Donglin'	93.700±28.760 fghi	63.174±6.876 def	45.862±4.065 ghij	8.465±1.277 jklm	15.526±7.335 cdef	15.865±1.728 kl	18.128±1.490 no	11.800±5.074 a	4.600±0.503 a
17	'Weixi Hong'	105.600±44.235 cdef	64.165±10.265 cde	47.059±7.567 efg	10.077±2.075 efg	14.822±7.305 cdefgh	19.301±1.939 bcdef	21.777±3.096 defg	5.150±2.700 ghi	3.100±0.968 efghijk
18	'Lifang'	123.323±29.229 bc	68.634±6.532 abc	57.618±6.218 ab	9.715±1.465 fgh	18.678±6.669 bc	14.315±2.064 m	20.973±2.321 efghijk	8.050±3.103 de	3.250±0.550 defghij
19	'Mudankui'	59.929±20.068 klmno	51.071±6.724 lmno	42.723±5.642 jk	7.384±1.042 nopqr	10.190±5.071 ijklmnop	14.858±2.146 lm	18.544±2.291 lmno	4.250±2.314 ghijkl	2.750±0.550 ijkl
20	'Dahongpao'	52.628±26.114 nopqr	52.963±7.993 jklmn	39.658±5.630 kl	5.254±0.858 uv	9.160±6.163 jklmnopqr	16.896±2.086 hijk	19.707±2.149 jklm	3.950±2.605 ghijkl	2.750±0.786 ijkl
21	'Chudie'	75.054±30.991 ijkl	60.260±8.562 efgh	47.001±4.922 efgh	9.521±1.505 ghi	6.172±3.162 pqrs	19.748±1.843 abcd	17.498±1.913 op	2.000±1.124 mno	2.550±0.826 klm
22	'Yulan Cha'	71.452±14.204 jklmn	54.883±3.774 ijkl	43.063±3.323 ijk	7.602±0.740 mnopq	16.226±4.962 cdef	17.146±1.869 hijk	21.182±1.307 efghijk	7.050±2.282 ef	3.350±0.587 defgh
23	'Manwu'	58.234±25.709 klmno	51.328±5.521 lmno	40.079±5.973 kl	6.204±1.069 st	9.716±6.051 ijklmnopq	17.829±1.507 fghij	19.907±1.306 ijkl	3.850±2.084 hijklm	2.350±0.671 lmn
24	'Xianye Cha'	46.807±17.833 opqrs	51.232±6.309 lmno	34.232±4.311 n	7.151±1.326 pqr	8.895±5.460 jklmnopqr	17.272±1.467 ghijk	20.292±1.510 ghijk	3.400±1.847 ijklmn	2.650±1.040 kl
25	'Lianrui'	45.340±28.246 opqrs	47.809±9.350 opq	36.200±5.966 mn	6.553±1.513 rst	8.338±7.116 klmnopqr	16.798±3.927 ijk	20.599±2.469 fghijk	3.300±3.230 ijklmno	1.900±0.912 n
26	'Duxinshizitou'	33.114±8.498 rs	43.253±4.697 qr	35.752±3.502 mn	7.708±1.224 lmnopq	3.517±1.673 s	17.312±1.828 ghijk	19.542±2.083 klmn	1.450±0.759 o	1.350±0.745 o

Continued Table S1

No.	Cultivars	FW	FH	FL	PT	SW	SL	SH	SN	ON
27	'Jiaohe'	36.215±11.822 pqrs	46.105±7.074 pq	32.842±4.100 n	7.303±1.154 opqr	4.972±1.764 rs	15.815±1.712 kl	18.233±1.716 mno	2.600±0.940 lmno	2.400±0.940 lmn
28	'Dandinghe'	69.969±27.000 jklmn	58.781±9.900 fghi	40.434±5.692 kl	8.189±1.392 jklmno	11.804±6.352 ghijkl	17.782±2.549 fghij	20.651±2.109 fghijk	5.100±2.100 ghij	2.850±0.875 ghijkl
29	'*C. reticulata* Ⅰ'	54.428±20.097 mnopq	52.198±6.832 klmno	38.709±4.437 lm	7.910±1.178 klmnop	5.250±2.868 qrs	18.376±1.999 defghi	21.989±5.313 cdef	1.650±0.933 no	2.000±0.918 n
30	'*C. reticulata* Ⅱ'	73.356±19.655 ijklm	51.512±4.439 lmno	54.575±4.869 bc	8.833±1.430 hijk	12.396±3.443 fghijk	18.814±1.459 cdefg	23.302±1.664 abcd	4.600±2.162 ghijk	3.650±0.671 kl
31	Zixi Mountain Ⅰ	78.588±23.005 ijk	57.415±5.010 ghij	45.732±5.138 ghij	12.410±1.798 b	9.021±3.290 jklmnopqr	16.755±2.383 jk	22.027±2.187 cdef	3.000±1.170 klmno	3.100±0.718 efghijk
32	Zixi Mountain Ⅱ	114.933±16.777 bcde	64.757±3.426 cde	46.280±3.212 fghij	10.610±0.747 def	22.232±4.459 b	17.236±1.333 ghijk	21.987±1.764 cdef	10.050±1.986 bc	4.400±0.503 ab
33	Hemu Village Ⅰ	114.051±28.431 cde	64.780±5.952 cde	50.839±4.952 de	11.300±1.248 cd	26.857±8.416 a	17.845±1.678 fghij	22.503±2.771 bcde	10.200±3.172 abc	3.350±0.671 defgh
34	Hemu Village Ⅱ	52.861±9.627 nopqr	49.728±3.124 mnop	40.442±4.740 kl	8.302±1.813 jklmno	11.005±3.642 ghijklmno	17.682±2.942 ghij	20.643±2.583 fghijk	5.000±2.534 ghij	2.800±0.768 hijkl
35	Fengqing Ⅰ	149.068±33.871 a	68.910±6.133 abc	60.534±5.863 a	16.115±1.411 a	15.189±7.118 cdefg	18.257±2.897 efghij	22.580±1.851 bcde	5.450±2.892 fgh	2.750±0.444 ijkl
36	Yangbi Ⅰ	47.794±9.752 opqrs	48.688±4.114 nop	34.313±3.151 n	7.631±0.861 mnopq	7.926±2.419 klmnopqr	13.432±1.557 mn	15.522±1.576 qr	8.000±1.686 de	3.300±0.470 defghi
37	Yongping Ⅰ	28.499±4.713 s	41.032±2.077 r	26.654±1.346 o	4.723±0.408 v	6.493±1.408 opqrs	12.653±1.109 n	15.052±1.192 r	8.900±1.586 cd	3.350±0.489 defgh
38	Heiniu Mountain Ⅰ	73.714±20.503 ijklm	54.258±5.383 ijklm	47.607±3.146 efg	8.692±1.077 ijkl	12.335±2.957 fghijk	14.605±1.854 lm	18.115±2.251 no	10.950±2.395 ab	3.250±0.444 defghij
39	Heiniu Mountain Ⅱ	83.133±12.806 hij	61.075±5.496 efg	43.232±2.908 hijk	11.115±0.879 cd	7.695±2.278 lmnopqrs	13.910±1.460 mn	16.510±1.611 pq	7.750±2.221 de	3.100±0.447 efghijk

FW: Fruit Weight; FL: Fruit Transverse Diameter; FH: Fruit Longitudinal Diameter; PT: Peel Thickness; SW: Seed Fresh Weight; SL: Seed Transverse Diameter; SH: Seed Longitudinal Diameter; SN: Seed Number; ON: The Number of Locules. Completely different lowercase letters indicate a significant difference at $P < 0.05$.

Table S2. Analysis of fruit color phenotypic differences between *C. reticulata* populations and its cultivars.

No.	Cultivars	*L**	*a**	*b**	*h°*	*C**
1	'Manao'	44.313±2.945 lmnop	7.125±0.764 op	26.282±1.452 opq	1.306±0.027 abc	27.239±1.468 rt
2	'Jinpaohong'	42.458±1.857 opqr	7.117±0.423 op	25.389±1.509 pqr	1.297±0.024 abc	26.376±1.426 tu
3	'Maye Yinhong'	45.408±3.344 jklmn	10.042±1.760 defghij	31.366±2.831 efghi	1.261±0.045 abc	32.968±2.966 hijk
4	'Dali Cha'	44.017±2.523 mnop	7.048±0.816 op	27.685±0.980 lmnop	1.322±0.032 abc	28.583±0.886 qrst
5	'Xuejiao'	44.914±1.711 klmno	13.490±1.428 a	31.421±1.827 efghi	1.165±0.041 c	34.218±1.911 ghi
6	'Dali Diechi'	42.012±1.295 pqr	7.387±0.847 opq	26.534±1.193 nopq	1.299±0.036 abc	27.560±1.098 str
7	'Fengshan Cha'	40.017±3.640 rs	10.220±1.565 cdefgh	24.604±4.019 qr	1.173±0.059 c	26.681±4.046 t
8	'Seben'	43.114±3.210 nopq	10.373±1.067 bcdefg	26.340±3.158 opq	1.192±0.055 bc	28.347±2.964 qrst
9	'Meihong Guiye'	47.394±3.836 defghijk	6.752±1.004 pq	29.276±1.729 hijklm	1.342±0.042 abc	30.071±1.531 nopq
10	'Shizitou'	40.946±2.439 qrs	6.570±0.593 pq	25.973±0.811 opq	1.323±0.022 abc	26.797±0.804 t
11	'Jingan Cha'	38.748±2.545 s	7.335±0.756 opq	23.271±3.721 r	1.257±0.074 abc	24.454±3.403 u
12	'Kunmingchun'	44.853±1.646 klmno	8.261±0.780 lmno	28.227±1.704 klmno	1.284±0.039 abc	29.432±1.494 pqrs
13	'Zaotaohong'	48.490±2.686 cdefgh	8.799±1.133 ijklm	30.925±3.410 efghij	1.293±0.027 abc	32.164±3.488 ijklmn
14	'Zhichun 08'	48.739±2.807 cdefg	10.820±1.903 bcdef	35.485±3.555 bc	1.275±0.047 abc	37.136±3.641 bcd
15	'Lichan'	47.942±3.391 cdefghij	10.115±2.764 cdefghi	35.355±3.941 bc	1.288±0.090 abc	36.921±3.445 bcde
16	'Donglin'	46.052±2.776 ghijklm	9.256±1.501 ghijklm	29.574±3.667 hijklm	1.262±0.079 abc	31.074±3.174 klmnop
17	'Weixi Hong'	44.028±1.503 mnop	9.392±1.118 ghijkl	28.015±1.893 lmno	1.248±0.024 abc	29.555±2.088 opqr
18	'Lifang'	50.179±4.063 bc	9.970±2.156 efghij	38.126±3.922 a	1.312±0.063 abc	39.480±3.742 a
19	'Mudankui'	46.097±2.166 fghijklm	9.274±1.399 ghijklm	28.740±3.875 jklmn	1.254±0.069 abc	30.266±3.565 nopq
20	'Dahongpao'	48.984±3.533 cde	8.539±1.285 klmn	30.705±3.061 fghij	1.297±0.053 abc	31.911±2.861 jklmn
21	'Chudie'	49.240±2.617 cd	5.763±1.228 q	30.680±2.064 fghij	1.384±0.044 a	31.247±1.966 klmnop
22	'Yulan Cha'	51.825±3.945 b	8.883±0.836 hijklm	32.940±2.400 def	1.306±0.035 abc	34.135±2.281 ghij
23	'Manwu'	48.849±3.815 cdef	8.711±1.312 jklm	31.580±1.931 efgh	1.302±0.047 abc	32.791±1.801 hijkl
24	'Xianye Cha'	46.844±4.319 defghijkl	9.949±0.824 efghij	31.043±3.472 efghij	1.258±0.043 abc	32.624±3.321 hijklm
25	'Lianrui'	49.364±4.111 cd	8.135±0.849 lmno	29.481±2.339 hijklm	1.299±0.042 abc	30.607±2.152 lmnopq

Continued Table S2

No.	Cultivars	*L**	*a**	*b**	*h°*	*C**
26	'Duxinshizitou'	48.990±4.798 cde	11.511±2.180 b	33.362±3.674 cde	1.237±0.057 abc	35.346±3.771 defg
27	'Jiaohe'	46.372±2.816 efghijklm	9.299±0.701 ghijklm	30.014±2.395 ghijkl	1.270±0.028 abc	31.433±2.342 klmnop
28	'Dandinghe'	45.896±3.831 hijklm	11.201±2.340 bcde	30.770±4.796 fghij	1.217±0.079 abc	32.828±4.758 hijkl
29	'*C. reticulata* Ⅰ'	48.211±6.620 cdefghi	9.066±2.698 ghijklm	31.407±3.095 efghi	1.292±0.072 abc	32.775±3.317 hijkl
30	'*C. reticulata* Ⅱ'	44.575±2.845 lmnop	6.613±1.482 pq	31.085±2.771 efghij	1.279±0.051 ab	31.819±2.075 klmno
31	Zixi Mountain Ⅰ	46.682±1.446 defghijklm	9.266±0.953 ghijklm	30.419±1.110 ghijk	1.275±0.035 abc	31.816±0.981 klmno
32	Zixi Mountain Ⅱ	46.632±3.633 defghijklm	9.083±1.066 ghijklm	29.018±3.482 ijklm	1.265±0.044 abc	30.431±3.419 mnopq
33	Hemu Village Ⅰ	47.757±4.464 cdefghij	8.267±1.471 lmno	33.279±4.326 cde	1.323±0.056 abc	34.339±4.158 fghi
34	Hemu Village Ⅱ	48.826±3.879 cdef	11.358±2.414 bc	32.417±3.560 defg	1.235±0.057 abc	34.405±3.809 fghi
35	Fengqing Ⅰ	45.735±2.775 ijklmn	8.544±1.111 klmn	27.200±2.249 mnop	1.266±0.040 abc	28.531±2.253 qrst
36	Yangbi Ⅰ	52.285±5.305 b	8.027±2.016 mno	36.736±6.239 ab	1.340±0.137 abc	37.801±5.215 abc
37	Yongping Ⅰ	46.866±4.703 defghijkl	9.728±3.701 fghijk	33.255±5.079 cde	1.280±0.118 abc	34.883±4.718 efgh
38	Heiniu Mountain Ⅰ	66.594±4.703 a	−1.531±4.970 r	38.465±5.434 a	−0.611±1.359 d	38.925±4.393 ab
39	Heiniu Mountain Ⅱ	50.210±4.857 bc	11.308±2.521 bcd	34.606±4.624 bcd	1.255±0.048 abc	36.449±4.953 cdef

Completely different lowercase letters indicate a significant difference, with a significant level of 0.05.

Table S3. Principal component analysis of fruit phenotypic traits in different populations and cultivars of *C. reticulata.*

**Traits(eigenvectors)**	**Principal component analysis of populations**		**Principal component analysis of cultivars**	
	**PC1**	**PC2**	**PC1**	**PC2**
FW	0.944	0.175	0.962	0.150
FL	0.923	0.154	0.912	0.690
FH	0.915	-0.30	0.904	-0.153
PT	0.836	-0.233	0.833	0.169
SW	0.797	-0.253	0.736	-0.518
SL	0.721	0.491	0.685	0.700
SH	0.716	-0.315	0.666	-0.423
SN	-0.033	0.912	0.263	0.815
ON	0.026	0.742	0.506	0.634
Eigenvalue	4.948	1.898	5.047	1.574
Contribution rate/%	54.979	21.086	56.077	17.493
Cumulative contribution rate/%	54.979	76.064	56.077	73.570

FW: Fruit Weight; FL: Fruit Transverse Diameter; FH: Fruit Longitudinal Diameter; PT: Peel Thickness; SW: Seed Fresh Weight; SL: Seed Transverse Diameter; SH: Seed Longitudinal Diameter; SN: Seed Number; ON: The Number of Locules.

Table S4. Analysis of various morphological traits of different populations and cultivars of *C. reticulata.*

Fruit phenotypic traits	*CV* （Mean±SD）/%		
	I	II	III
FW	65.436±29.902	113.631±37.501	38.146±12.354
	45.696	33.003	32.385
FL	54.278±8.770	67.068±7.669	44.860±5.038
	16.157	11.435	11.230
FH	42.432±7.246	50.674±7.305	30.484±4.557
	17.075	14.415	14.948
PT	8.174±2.177	10.494±2.461	6.177±1.616
	26.637	23.447	26.163
SW	9.903±6.124	18.236±9.126	7.210±2.084
	61.833	50.046	28.908
SL	17.288±2.608	18.816±2.712	13.043±1.392
	15.085	14.411	10.669
SH	20.183±2.869	22.768±2.381	15.287±1.400
	14.216	10.456	9.158
SN	4.658±3.490	6.338±3.668	8.450±1.679
	74.917	57.879	19.866
ON	2.820±0.999	3.396±0.890	3.325±0.474
	35.418	26.214	14.266
Mean /%	34.115	26.812	18.621

FW: Fruit Weight; FL: Fruit Transverse Diameter; FH: Fruit Longitudinal Diameter; PT: Peel Thickness; SW: Seed Fresh Weight; SL: Seed Transverse Diameter; SH: Seed Longitudinal Diameter; SN: Seed Number; ON: The Number of Locules.

Table S5. Frequency distribution function of fruit phenotypic traits of different populations and cultivars of *C. reticulata.*

Fruit phenotypic traits		Frequency distribution function			
		**CV**	**R^2**	**WT**	**R^2**
Quality characteristics	FW	$y=0.807+18.902e^{-0.0003(x-64.108)^2}$	0.875	$y=0.041+18.836e^{-0.0002(x-62.535)^2}$	0.952
	SW	$y=1.629+27.366e^{-0.0124(x-8.746)^2}$	0.952	$y=3.863+29.445e^{-0.0506(x-8.948)^2}$	0.925
Dimensional characteristics	FL	$y=-2.239+21.977e^{-0.0026(x-57.396)^2}$	0.963	$y=-37.251+53.428e^{-0.0006(x-56.859)^2}$	0.784
	FH	$y=-0.548+22.683e^{-0.0056(x-43.860)^2}$	0.977	$y=2.589+17.748e^{-0.0077(x-44.607)^2}$	0.793
	PT	$y=-0.119+36.353e^{-0.1011(x-8.135)^2}$	0.990	$y=2.461+25.508e^{-0.0961(x-9.366)^2}$	0.932
	SL	$y=0.387+45.591e^{-0.0791(x-18.359)^2}$	0.980	$y=0.164+25.957e^{-0.0497(x-15.167)^2}$	0.933
	SH	$y=0.143+41.841e^{-0.0631(x-20.973)^2}$	0.999	$y=-3.128+21.997e^{-0.0192(x-18.860)^2}$	0.819
Quantitative characteristics	SN	$y=1.582+46.506e^{-0.1107(x-4.068)^2}$	0.975	$y=-0.018+25.084e^{-0.0492(x-8.914)^2}$	0.970
	ON	$y=5.675+38.314e^{-192.234(x-3.240)^2}$	0.673	$y=3.734+59.209e^{-102.041(x-3.194)^2}$	0.853
